# In vivo expression of anti-CD19/CD3 BiTE by liver-targeted AAV for the treatment of B cell malignancies

**DOI:** 10.1038/s41408-024-01036-4

**Published:** 2024-03-15

**Authors:** Zhiqiang Song, Ping Liu, Dongliang Zhang, Tao Wang, Wenqin Yue, Yuke Geng, Na Liu, Yang Wang, Jianmin Yang

**Affiliations:** Department of Hematology, Institute of Hematology, Changhai Hospital, Naval Medical University, 200433 Shanghai, China

**Keywords:** B-cell lymphoma, Immunotherapy, Targeted therapies, Acute lymphocytic leukaemia

Dear Editor,

Anti-CD19/CD3 bispecific T-cell engagers (CD19BiTE) have shown promising efficacy in patients with relapsed or refractory (r/r) B-cell acute lymphoblastic leukemia (B-ALL) and diffuse large B-cell lymphoma (DLBCL) [1, 2]. However, the half-life of blinatumomab in patients is about 2.1 h and continuous administration over one cycle of 4 weeks is indispensable [3, 4]. In addition, short half-life and intermittent breaks during the therapy make the drug concentration unstable and the anti-leukemia effect could be achieved when dosage reaches 28 μg/d [5–7]. Consequently, the short half-life of blinatumomab not only hurdles the widespread usage in the clinic but also compromises its efficacy.

To enhance the efficacy and avoid continuous infusion, in vivo expression of CD19BiTE may be a promising strategy. Adeno-associated virus (AAV) has been widely used in preclinical studies and clinical trials due to its wide host range, high safety, low immunogenicity, and stable expression [8, 9]. Previous studies have proved that AAV8 has strong liver tropism and high infection efficiency, and AAV8 is simple and feasible for large-scale preparation, which contributes to widespread clinical application [10, 11]. Here, we created recombinant AAV8 encoding CD19BiTE (AAV-CD19BiTE) to achieve sustained expression of CD19BiTE in vivo. Meanwhile, we integrated the liver-specific promoter thyroxine-binding globulin (TBG) into the AAV8 sequences in order to reduce the potential adverse effects of systemic expressions, such as central nervous system toxicity and cardiotoxicity [12, 13].

Firstly, we constructed the recombinant AAV encoding CD19BiTE according to the previous study [14]. The schematic of recombinant AAV expressing CD19BiTE (AAV-CD19BiTE) was presented in Fig. S1A. CD19BiTE sequence was composed of CD19 and CD3 single-chain fragment variables, linker sequence and His-Tag sequences. Next, AAV-CD19BiTE was used to transfect the 293T, HepG2 and PLC/PRF/5 cells, of which HepG2 and PLC/PRF/5 cells are human hepatoma cell lines. The anti-CD3 and CD19 binding competition assays indicated that HepG2 and PLC/PRF/5 cells could secret the CD19BiTE, while 293T cells failed to produce CD19BiTE (Fig. S1B). Further, His-Tag immunofluorescence analysis was performed to prove the expression of CD19BiTE. As shown in Fig. S1C, the fluorescence of transfected 293T cells was unable to observe but it was obvious in transfected HepG2 cells. These results indicated that recombinant AAV-CD19BiTE was liver-targeted.

Next, we analyzed the CD19-specific tumor-kill ability of secreted CD19BiTE in vitro. CD107a is a sensitive marker to determine the cytotoxic activity of CD8^+^T cells [15]. The supernatants of 293T, HepG2 and PLC/PRF/5 cells were co-cultured with PBMC and CD19^+^NALM-6 cells for 4 h. Then, we evaluated the CD8^+^CD107a^+^ ratios of various co-culture systems, and only the supernatants of HepG2 and PLC/PRF/5 cells were capable of stimulating CD8^+^T cells to perform degranulation-killing activity (Fig. S1D, E). Cytotoxicity of AAV-CD19BiTE was further analyzed in CD19^+^NALM-6 and Raji cells, and CD19^−^K562 cells. Robust cytotoxicity was observed in NALM-6 and Raji cells rather than K562 cells (Fig. S1F), indicating the antitumor activity of secreted CD19BiTE depended on CD19 expression. Meanwhile, the contents of IL-2, TNF-α, and IFN-γ were significantly higher in co-culture mediums of NALM-6 and Raji’s cells mixed with AAV-CD19BiTE compared to AAV-GFP, while no obvious changes were observed in the co-culture medium of K562 cells (Fig. S1G). These results showed that AAV-CD19BiTE could specifically kill CD19^+^ tumor cells.

We further evaluated the expression and sustained expression time of AAV-CD19BiTE in vivo. The RT-qPCR analysis of multiple organs suggested that CD19BiTE could only be expressed in the liver (Fig. S2A), consistent with in vitro results. Meanwhile, the serum of mice was able to activate T cells (Fig. S2B), suggesting the successful secretion of CD19BiTE in vivo. In addition, we traced the changes in the contents of CD19BiTE in vivo by collecting the serum of mice once or twice a week. The peak level of CD19BiTE, reached at 4 weeks after AAV injection, was about 2500 pg/mL. Of note, it could achieve stable expression for more than half a year (Fig. S2C).

After proving the antitumor activity of AAV-CD19BiTE in vitro and its sustainable expression in vivo, we continued to explore the anti-leukemia activity of AAV-CD19BiTE in vivo. The NCG mice were randomly divided into PBMC, PBMC+AAV-GFP (AAV-GFP), and PBMC+AAV-CD19BiTE (AAV-CD19BiTE) groups (*n* = 5). The tumor burdens of mice in PBMC and AAV-GFP groups increased rapidly, and all mice died due to high tumor burdens at day 21 following NALM-6 cells injection (Fig. 1A). Meanwhile, the mice’s weights of these two groups began to decrease continuously after 1 week of NALM-6 cells injection (Fig. 1B). Conversely, the treatment of AAV-CD19BiTE could effectively reduce the tumor burdens, and there was no significant weight loss in mice of AAV-CD19BiTE group. On day 19 after NALM-6 cells injection, the mice’s tumor burdens of the CD19BiTE group were significantly lower than those of PBMC and AAV-GFP groups (Fig. 1C). In addition, the survival was greatly prolonged after the treatment of AAV-CD19BiTE compared to the other two groups (Fig. 1D). AAV-CD19BiTE exhibited robust anti-leukemia effects in vivo.

**Fig. 1:**
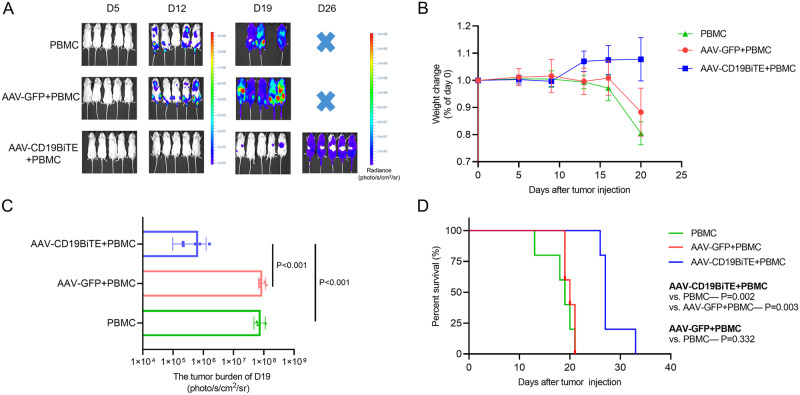
Anti-leukemia activity of AAV-CD19BiTE in vivo. **A** NCG mice were intravenously injected with 2 × 10^6^ NALM-6 cells, followed by infused with PBMC, PBMC+AAV-GFP (AAV-GFP), and PBMC+AAV-CD19BiTE (AAV-CD19BiTE), respectively. Bioluminescent images of differently treated mice over time (*n* = 5). **B** The weight changes of differently treated mice. **C** The comparison of tumor burdens on day 19 following NALM-6 cells injection. *P* values were calculated using a one-way ANOVA test with post-hoc analysis. **D** Kaplan–Meier analysis was performed to evaluate the survival differences among different treatment groups.

We next investigated the anti-lymphoma activity of AAV-CD19BiTE in vivo by establishing a B-cell non-Hodgkin’s lymphoma model and infusing it with AAV-CD19BiTE. Although the mice’s tumor burdens of AAV-CD19BiTE group were obviously higher than those of PBS and AAV-GFP groups at the beginning of treatment, the tumor burdens increased slower after 2 weeks and began to decrease after 3 weeks of AAV-CD19BiTE injection (Fig. 2A). Meanwhile, the contents of TNF-α and IFN-γ in AAV-CD19BiTE group were significantly higher than PBS and AAV-GFP groups (Fig. 2B). Consistent with the results of bioluminescent imaging, the tumor volumes began to reduce after 3 weeks of AAV-CD19BiTE injection, while tumor volumes of the other two groups increased rapidly (Fig. 2C). Of note, 2 of 5 mice achieved CR following AAV-CD19BiTE therapy. The treatment with AAV-CD19BiTE could significantly inhibit the growth of Raji tumor cells and prolong the survival of mice (Fig. 2D, E). To better understand the anti-lymphoma effect of AAV-CD19BiTE, we further performed tumor microenvironment analyses. Flow cytometry analysis revealed that the contents of CD3^+^ and CD8^+^T cells in the AAV-CD19BiTE group were significantly higher than the AAV-GFP group (Fig. S3A, B). Similarly, more CD3^+^, CD4^+^, and CD8^+^T cells were also observed following AAV-CD19BiTE therapy in immunohistochemistry analysis (Fig. S3C, D). Of note, immunofluorescence analysis suggested that the levels of activated CD8^+^T cells (CD8^+^CD69^+^) were notably higher after AAV-CD19BiTE infusion compared to AAV-GFP (Fig. S3E, F). AAV-CD19BiTE was capable of recruiting and activating more immune cells to kill the lymphoma.

**Fig. 2:**
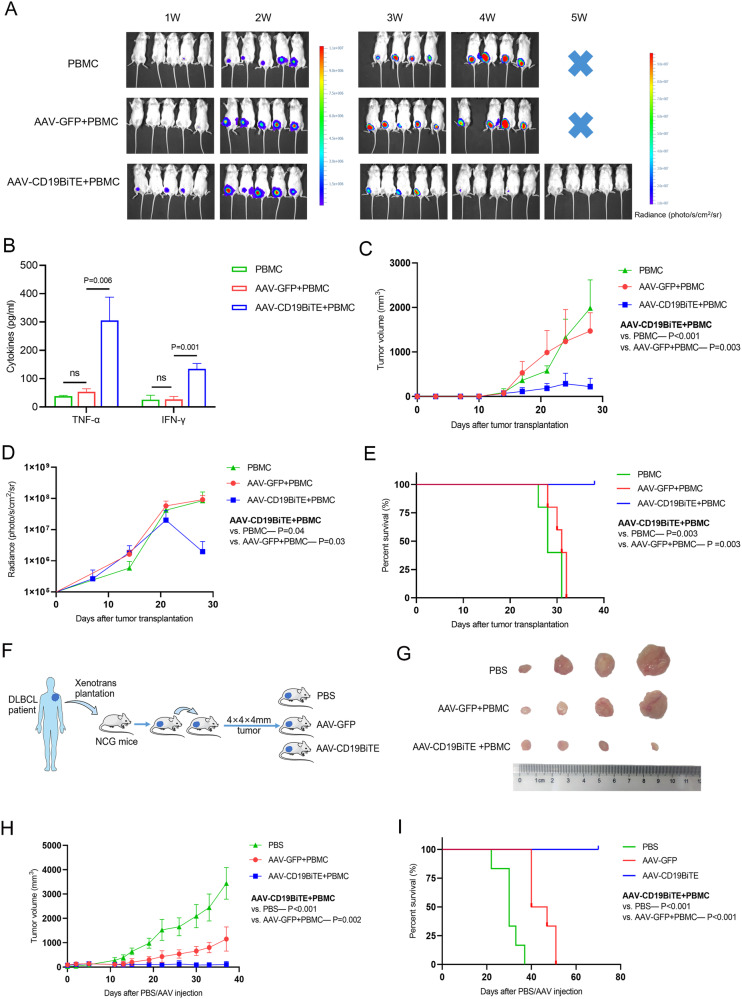
Anti-lymphoma activity of AAV-CD19BiTE in CDX and PDX model. **A** NCG mice were subcutaneously transplanted with 2 × 10^6^ Raji cells, followed by infused with PBMC, PBMC+AAV-GFP (AAV-GFP), and PBMC+AAV-CD19BiTE (AAV-CD19BiTE), respectively. Bioluminescent images of differently treated mice over time (*n* = 5). **B** The contents of TNF-α and IFN-γ in different treatment mice. **C** The changes in tumor volumes among different treatment groups. **D** The changes of radiance values among different treatment mice. **E** Kaplan–Meier analysis was performed to evaluate the survival differences between different treatment mice. **F** Schematics of constructed PDX model deriving from DLBCL patient tumor tissues. **G** The changes in tumor sizes among different treatment groups (*n* = 6). **H** The changes in tumor volumes among different treatment groups. **I** Kaplan–Meier analysis was performed to analyze the survival differences between different treatment groups. *P* values were calculated using a one-way ANOVA test with post-hoc analysis except survival analyses.

Further, to better mimic the tumor microenvironment of B-cell non-Hodgkin’s lymphoma, we constructed the DLBCL-PDX model (Fig. 2F). After establishing the PDX model successfully (Fig. S4), the NCG mice were randomly divided into PBS, PBMC + AAV-GFP (AAV-GFP), and PBMC+AAV-CD19BiTE (AAV-CD19BiTE) groups (*n* = 6). The mice’s tumor volumes increased continuously and quickly in PBS and AAV-GFP groups, while tumor volumes of the AAV-CD19BiTE group began to decline after a slight increase (Fig. 2G, H). In the long-term observation, 4 of 6 mice achieved CR following AAV-CD19BiTE infusion. Meanwhile, the mice treated with AAV-CD19BiTE survived longer than PBS and AAV-GFP (Fig. 2I). Although the growth of tumor volumes was slightly slower in mice of AAV-GFP group compared to the PBS group in a short time, the tumor volumes still continued to increase until similar to PBS group in a long-term observation (Fig. S5A). Of note, even though the tumor burdens were notably high (the diameter was more than 8 mm) at the beginning of treatment, AAV-CD19BiTE still could reduce the tumor volume until CR(Fig. S5B). Potent anti-lymphoma activity of AAV-CD19BiTE was also observed in the DLBCL-PDX model.

Finally, we analyzed the potential toxicity of AAV-CD19BiTE in vivo (Fig. S6A). The weights of mice increased gradually following the injection of AAV-CD19BiTE (Fig. S6B). Meanwhile, the blood cell counts were normal and similar to mice treated with PBS (Fig. S6C). Multiple serum biochemical indicators were also normal, and there were no significant differences between PBS and AAV-CD19BiTE groups (Fig. S6D). Multiple tissue section analyses suggested no obvious changes following AAV-CD19BiTE injection (Fig. S6E). Additionally, 23 cytokines were measured using Luminex, and no significant changes were observed between PBS and AAV-CD19BiTE groups (Fig. S6F). Collectively, these results proved the safety of AAV-CD19BiTE in vivo.

In summary, this study provided a proof of concept that liver-targeted AAV encoding the CD19BiTE could achieve the long-term and stable expression of CD19BiTE in vivo. Furthermore, in vitro and in vivo experiments have proved its potent therapeutical effect on B-cell malignancies. This new treatment strategy is expected to reduce the costs of blinatumomab and improve the efficacy of the treatment of r/r B-cell malignancies.

### Supplementary information

Supplementary materials

## Data Availability

The data that support the findings of this study are available from the corresponding authors upon reasonable request.
