# Supplementary material for: In vivo expression of anti-CD19/CD3 BiTE by liver-targeted AAV for the treatment of B cell malignancies
Source: Blood Cancer J. 2024 Mar 15;14(1):46. doi: 10.1038/s41408-024-01036-4 (PMC10940594; doi:10.1038/s41408-024-01036-4)
Supplement: Supplementary file 1 — Supplementary materials [file 41408_2024_1036_MOESM1_ESM.docx]

**In vivo expression of** **anti-CD19/CD3 BiTE by** **liver-targeted AAV for the treatment of B cell malignancies**

**Supplementary Materials and Methods:**

**Cells and cell culture**

293T, NALM-6, Raji, Jurkat, and K562 cell lines were provided by cell bank of Department of Hematology, Changhai Hospital. HepG2 and PLC/PRF/5 cell lines were gifts from Dr. Sun (The Third Affiliated Hospital of Naval Medical University). NALM-6, Raji, Jurkat, and K562 cell lines were cultured in RPMI 1640 supplemented with 10% fetal bovine serum (FBS), 1% penicillin, and 1% streptomycin. 293T, HepG2 and PLC/PRF/5 cell lines were maintained in DMEM supplemented with 10% FBS, 1% penicillin, and 1% streptomycin. All cell lines were incubated at 37 °C with 5% CO_2_. NALM-6/Raji-luciferase cell lines were constructed as previous study reported[1]. All cells used in this study were checked to ensure no mycoplasma contamination.

**Antibodies**

The sources of all the antibodies used in this study were presented as follows: PerCP Anti-Human CD3 antibody (BioLegend, Cat#317338); APC Anti-Human CD19-antibody (BioLegend, Cat#363006); PE-Cy7 Anti-Human CD19 antibody (BioLegend, Cat#302216); APC Anti-Human CD8 antibody (BD pharmingen, Cat#555369); APC Anti-Human CD69 antibody (BD pharmingen, Cat#555533); PE Anti-Human CD107a antibody (BD pharmingen, Cat#555801); Zombie Violetm Fixable Viability Kit (BioLegend, Cat#423114); PE/Dazzlem Anti-Human CD45 antibody (BioLegend, Cat#368530); Anti-CD3 Mouse mAb (Servicebio, Cat#GB12014); Anti-CD8 alpha Mouse mAb (Servicebio, Cat#GB12068); HRP conjugated Goat Anti-Mouse IgG (Servicebio, Cat#GB23301); HRP conjugated Goat Anti-Rabbit IgG (Servicebio, Cat#GB23303); Recombinant Anti-His Tag antibody (Servicebio, Cat#GB151255); Alexa Fluor® 488-conjugated Goat Anti-Rabbit IgG (Servicebio, Cat#GB25303); CD69 Antibody (D-3) (Santa Cruz Biotechnology, Cat#sc-373799); CD4 Antibody (MT310) (Santa Cruz Biotechnology, Cat#sc-19641); Cy3 conjugated Goat Anti-mouse IgG (Servicebio, Cat#GB21301); Anti-CD8 alpha Rabbit pAb (Servicebio, Cat#GB114123); Alexa Fluor® 488-conjugated Goat Anti-Rabbit IgG (Servicebio, Cat#GB25303); DAPI (Servicebio, Cat#G1012).

**Recombinant AAV construction and production**

Recombinant liver-targeted AAV encoding CD19BiTE (AAV-CD19BiTE) and GFP (AAV-GFP) were constructed and produced in Vector Builder. The viral titers of AAV-CD19BiTE and AAV-GFP were 1.31×10^13^gc/mL and 1.87×10^13^gc/mL, respectively. The vector builder IDs for AAV-CD19BiTE and AAV-GFP were VB230320-1499hmj and VB230323-1005gjb, respectively and all the detailed information can be searched on vectorbuilder.com.
**In vitro transfection and binding assays**

A total of 2×10^5^ 293T, HepG2 and PLC/PRF/5 cells were inoculated into 6-well plates and supplemented with 2 mL medium. After 24 h, aspirating the supernatant and adding 1mL fresh medium. Then, thawing AAV-CD19BiTE/GFP on ice and adding an appropriate amount of AAV based on the multiplicity of infection (MOI) of 2×10^5^. 12 h after infection, aspirating the supernatant and adding 2 mL fresh medium. Finally, collecting the supernatant for competition binding assays after 72 h.

A total of 1×10^5^ NALM-6 and Jurkat cells were inoculated into 96-well plates and supplemented with 100 μL medium. Then, adding 100 μL AAV-CD19BiTE transfected 293T, HepG2 and PLC/PRF/5 cell supernatants into the 96-well plates with 3 repetitions, respectively. Control group was added 100 μL AAV-GFP transfected supernatant. After incubation for 12 h, the NALM-6 and Jurkat cells were collected and incubated with hCD19-APC and hCD3-PerCp for 15 min at room temperature, respectively, followed by flowcytometry analysis (BD Biosciences, FACSAria™).

**His-Tag immunofluorescence analysis**

The recombinant AAV-CD19BiTE contained the His-Tag sequence and to further validate the secretion of CD19BiTE after transfection, a His-Tag immunofluorescence analysis was performed as previous reported[2]. Placing sterile coverslips into six-well plates before transfection and adding 293T or HepG2 cells into plates. Then, performing transfection as mentioned above. 72 h after transfection, aspirating the cell supernatant and adding PBS solution to wash twice, and adding 2ml 4% paraformaldehyde solution to each six-well plate for fixation. Next, permeabilizing the cells with 0.5% Triton X-100 for 20min and adding 3% BSA to block for 30 minutes. Shaking off the blocking solution gently and adding His-Tag primary antibody for incubation at 4℃ overnight. Finally, incubating with secondary antibody at room temperature for 50min. Cell nuclei were marked with 4,6-diamidino-2-phenylindole (DAPI) and using Fluorescent Microscopy (Nikon, Nikon Eclipse C1) to collect the images.

**CD107a degranulation assay**

To preliminary analyzed the antitumor activity of AAV-CD19BiTE in vitro, we performed the CD107a degranulation assay[3]. Firstly, peripheral blood of healthy volunteers was collected from Changhai Hospital and peripheral blood mononuclear cells (PBMC) were obtained by Ficoll density gradient centrifugation. Then, adding 50 μL PBMC (3×10^6^/mL) and NALM-6 cells separately into U-shaped 96-well plates (E:T=5:1). Next, adding 100 μL AAV-CD19BiTE transfected 293T, HepG2 and PLC/PRF/5 cell supernatants into the 96-well plates respectively. Meanwhile, 10 μL hCD107a-PE antibody were added into the co-culture medium and incubating for 4 h at 37 °C with 5% CO_2_. Finally, cells were collected and incubated with hCD3-PerCp and hCD8-APC for 15 min at room temperature without light, followed by flowcytometry analysis of CD8^+^CD107a^+^ ratios.

**Cytotoxicity assays**

To assess the targeted-kill ability of AAV-CD19BiTE in vitro, we firstly marked the K562, NALM-6, and Raji cells with carboxyfluorescein succinimidyl ester (CFSE) as previously reported[4]. Then, 50 μL PBMC (1×10^7^/mL) and 50 μL CFSE-labeled K562, NALM-6, and Raji cells were added into 96-well plates, followed by adding 100 μL AAV-CD19BiTE transfected HepG2 cell supernatants. After co-culture for 48 h, collecting the cells and staining with Fixable Viability Stain 450 (FVS450, BD Biosciences) for determining the CFSE^+^FVS450^+^ ratios. Meanwhile, the cell supernatants were harvested for cytokine assays.

**Enzyme-linked immunosorbent assay (ELISA)**

The co-culture cell supernatants of PBMC and tumor cells were collected to measure the contents of IL-2, TNF-α and IFN-γ via ELISA (Mlbio, Cat#Ml058063, Ml077385, and Ml077386) based on the manufacturing protocol. Each cytokine was measured for 3 replicates.

**In vivo expression of AAV-CD19BiTE**

Six female NOD-Prkdc(em26Cd52)il2rg(em26Cd22)/Nju (NCG) mice were randomly equally divided into two groups and injected with AAV-CD19BiTE/AAV-GFP via the tail vein at the dose of 5×10^12^gc/kg. After 4 weeks, the mice were euthanatized and the liver, heart, spleen, lung, kidney, and brain in the AAV-CD19BiTE group were collected, pestled, and filtered for extracting RNA. Total RNA was obtained by Fastagen RNA Isolation Kit (Shanghai Feijie Biotechnology Co., Ltd) according to the protocols. Then, analyzing the contents of CD19BiTE in different tissues via real-time quantitative polymerase chain reaction (RT-qPCR) as previously reported[1]. Primers (5′-3′) of CD19BiTE was CTACTGGATGAACTGGGTGAAG (forward) and CTTGAACTTGCCGTTGTAGTTG (reverse). At the same time of euthanatizing mice, collecting the serum of different groups to evaluate the T cell activation capacity of CD19BiTE. Then, adding 50 μL PBMC (1×10^7^/mL) and 50 μL NALM-6 cells (E:T=5:1) into U-shape 96-well plates. Next, 100 μL serum collected above was added into the 96-well plates and incubated for 12 h. Finally, collected the cells and stained with hCD3 and hCD69 antibody for 15 min at room temperature, followed by flowcytometry analysis of CD3^+^CD69^+^ratios.

In order to trace the changes of CD19BiTE in vivo, 3 mice were injected with AAV-CD19BiTE (5×10^12^gc/kg) and tail vein serum was collected once a or two weeks for half a year. Then, the serum was frozen at -80℃ until all the samples were collected. Finally, measuring the levels of CD19BiTE via His-tag ELISA Detection Kit (GenScript, L00436) according to the manufacture protocols.

**Cell lines-derived xenograft (CDX) mice models of** **B-cell malignancies**

To validate the antitumor activity of AAV-CD19BiTE in vivo, 6- to 8-week-old, female NCG mice were fed in specific pathogen free (SPF) house and randomly divided into 3 groups: PBMC group, PBMC+AAV-GFP (AAV-GFP), and PBMC+AAV-CD19BiTE (AAV-CD19BiTE) groups using random number table method (n=5). 2×10^6^ NALM-6/Raji-luciferase cells were intravenously/subcutaneously implanted into NCG mice at day 1 to construct B-cell leukemia/lymphoma models. Then, AAV (5×10^12^gc/kg) was injected into AAV-GFP and AAV-CD19BiTE groups and mice of PBMC group were injected with equal volume of PBS at day 3, followed by 2×10^7^ PBMC injection via tail vein at day 5. For bioluminescent imaging in vivo, mice were intraperitoneally injected with 15 mg/mL D-luciferin potassium salt solution at the dose of 10 μL/g. 10 min after injection, the mice were anesthetized for bioluminescent imaging via VISQUE^®^ InVivo ART 100. Imaging was performed once a week and tumor burden is evaluated as photons per second per cm^2^ per steradian (photo/s/cm^2^/sr).

The survival of the mice was observed every day and weights were recorded every three days. Meanwhile, the tumor sizes of B-cell lymphoma were measured and calculated as follows: volume = length × width^2^ × 1/2. The mice were considered to be complete remission (CR) when tumor was not palpable and euthanized when the tumor volume exceeded 2000 mm^3^ or weight loss exceeded 20%. All the animal experiments were approved by the institutional review board of Changhai Hospital.

**Lymphoma microenvironment analyses of** **B-cell lymphoma**

To analyze the improvement of lymphoma microenvironment following AAV-CD19BiTE therapy, 2×10^6^ Raji-luciferase cells were implanted into right groin of NCG mice and mice were randomly divided into AAV-GFP and AAV-CD19BiTE groups on day 1 using random number table method (n=5). On day 3, AAV (5×10^12^gc/kg) was injected into mice of AAV-GFP and AAV-CD19BiTE groups, respectively, followed by 2×10^7^ PBMC injection via tail vein on day 5. 3 weeks after tumor cells injection, euthanizing the mice and collecting the tumors. For flowcytometry analysis, half of the lymphoma was dissociated into single-cell suspensions using gentleMACS™ Dissociator (Miltenyi,130-093-235). Then, single-cell suspensions were filtered using 70 μm membrane and washed twice with PBS. Next, single-cell suspensions were incubated with death dye, hCD45-PE-TexasRed, hCD3-PerCP, hCD4-FITC, and hCD8-APC for 15 min at room temperature, followed by flowcytometry analysis. For immunohistochemistry and immunofluorescence analyses, the remaining half of the lymphoma was fixed in 4% paraformaldehyde solution for more than 24 hours. Then, performing the immunohistochemistry analyses of CD3^+^, CD4^+^, and CD8^+^T cells contents in different groups as previously reported^[5]^. Meanwhile, performing the immunofluorescence analyses of CD8^+^CD69^+^T cells contents as previously reported[6]. The results were analyzed using Aipathwell artificial intelligence digital pathology image analysis software.

**Patient-derived xenograft (PDX) model of** **B-cell non-Hodgkin's lymphoma**

In order to further investigate the effects of AAV-CD19BiTE on B-cell non-Hodgkin's lymphoma, we established patient-derived xenograft (PDX) model by implanting the tumor tissues of primary diffuse large B cell lymphoma (DLBCL) patient in the right groin of NCG mice. After about 2 weeks, the tumors were palpable and the mice were randomly divided into 3 groups: PBS group, PBMC+AAV-GFP (AAV-GFP), and PBMC+AAV-CD19BiTE (AAV-CD19BiTE) groups using random number table method (n=6). Then, mice of AAV-GFP and AAV-CD19BiTE groups were injected with AAV-GFP and AAV-CD19BiTE (5×10^12^gc/kg), respectively. Meanwhile, mice of PBS group were injected with equal volume of PBS. 2 days after AAV injection, mice of AAV-GFP and AAV-CD19BiTE groups were intravenously infused with 1×10^7^ PBMC, while the PBS group also received equal PBS. Then, measuring the weights and tumor volumes once every three days. The mice were euthanized when the tumor volume exceeded 3000 mm^3^.

**Safety analysis**

6- to 8-week-old Balb/c mice were fed for acclimatization for one week and randomly divided into two groups, of which one was injected with AAV-CD19BiTE (5×10^12^gc/kg) and the other one received equal volume of PBS. Observing the mice every day and measuring the weights every three days. 4 weeks after injection, euthanizing the mice and collecting the liver, heart, spleen, lung, kidney, and brain tissues to perform hematoxylin and eosin analyses to observe the structures. Meanwhile, orbital blood was collected for blood counting analyses and serum was collected to measure various biochemical indications, such as ALT, AST, Cre, and CK-MB. In addition, serum was used to measure cytokines by Luminex 200 system (Luminex) according to protocols.

**Statistical analysis**

Continuous variables were presented as mean ± SD and comparisons were performed by unpaired, two tailed Student’s *t*-test or ANOVA for multiple comparisons using GraphPad Prism version 8.0. Homogeneity of variance between the groups was compared before statistical tests. The sample sizes were determined based on previous studies[7-9]. Survival evaluations were performed using Kaplan-Meier curves and Log-rank test. P<0.05 was considered to be significant.

**Supplementary Figures and Figure Legends:**

| **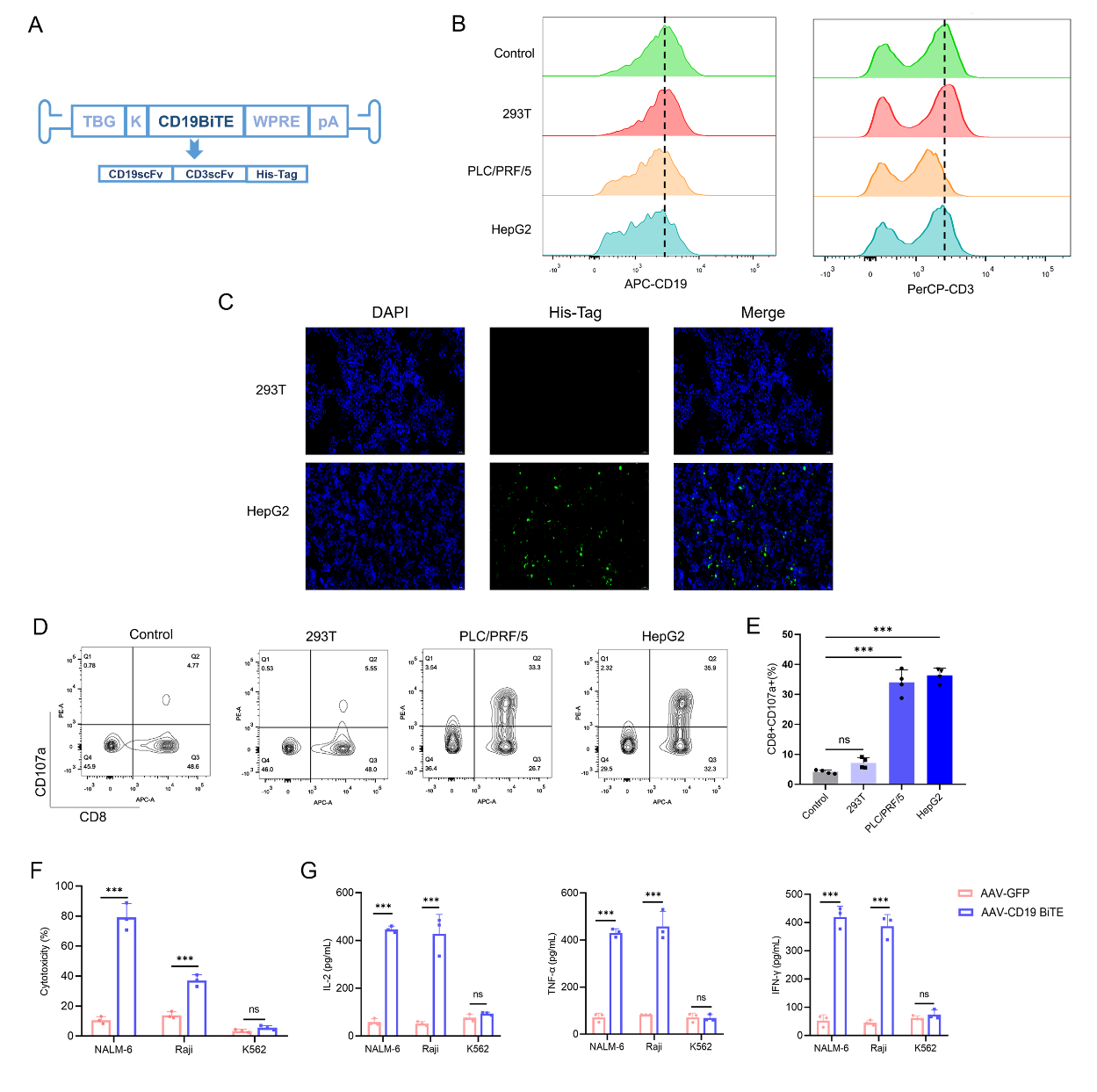** |
| --- |
| **Fig. S1.** **Construction and validation of** **recombinant AAV expressing** **CD19BiTE.** (A) Schematics of recombinant AAV expressing CD19BiTE plasmid. TBG, thyroxine binding globulin; K, Kozak sequence; WPRE, woodchuck hepatitis virus posttranscriptional regulatory element; pA, polyadenylation site; scFv, single-chain fragment variable. The CD19BiTE was composed of CD19 scFv, CD3 scFv, and His-Tag sequence. CD19 scFv and CD3 scFv were connected by a peptide linker. (B) The CD19 and CD3 competition binding assay. AAV-CD19BiTE transfected 293T, HepG2 and PLC/PRF/5 cell supernatants were co-cultured with NALM-6 and Jurkat cells for 12 h, and then we analyzed the CD19 and CD3 fluorescence intensity of NALM-6 and Jurkat cells, respectively. (C) His-Tag immunofluorescence analysis of AAV-CD19BiTE transfected 293T and HepG2 cells. (D) Flowcytometry analysis of CD107a^+^ ratios in CD8^+^ T cells. AAV-CD19BiTE transfected 293T, HepG2 and PLC/PRF/5 cell supernatants were co-cultured with PBMC and NALM-6 cells (E:T=5:1) for 4 h, then CD8^+^CD107a^+^ ratios were evaluated by flowcytometry. (E) The comparison of the ratios of CD8^+^CD107a^+^ cells after co-culture with different cell supernatants (n=4). (F) The cytotoxicity of AAV-CD19BiTE for CD19^+^ NALM-6 and Raji cells, and CD19^-^ K562 cells. AAV-CD19BiTE transfected HepG2 cell supernatant was co-cultured with PBMC and CFSE-stained tumor cells (E:T=5:1), respectively. After 48 h, the cells were collected and stained with Fixable Viability Stain 450 to analyze the CFSE^+^FVS450^+^ ratios (n=3). (G) Collecting the co-culture supernatants to analyze the contents of IL-2, TNF-α and IFN-γ via ELISA. *P < 0.05; **P < 0.01; ***P < 0.001, using two tailed Student’s *t*-test for two groups or one-way ANOVA test with post-hoc analysis for multiple groups. |

| **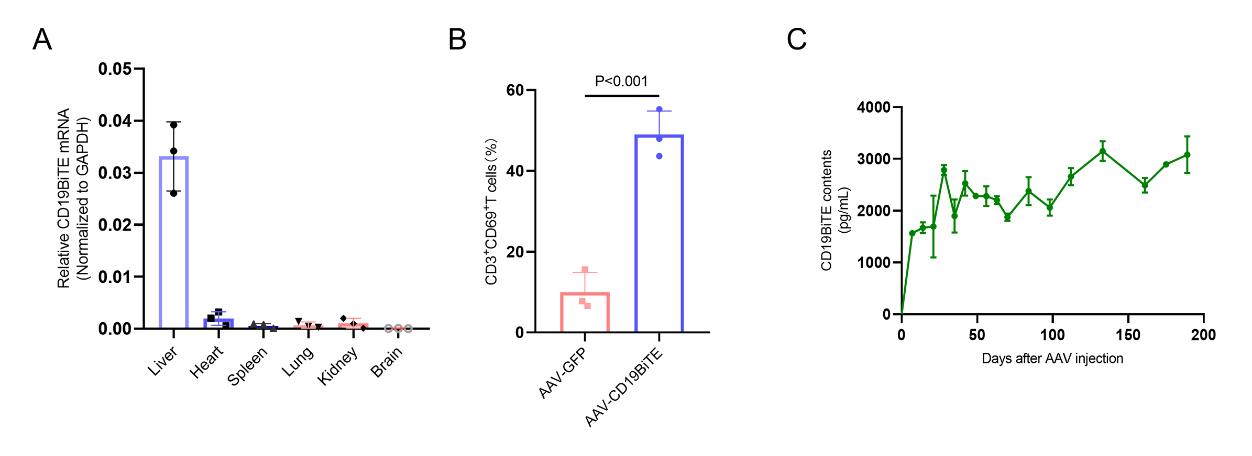** |
| --- |
| **Fig. S2. In vivo expression of AAV-CD19BiTE**. (A) NCG mice were injected with AAV-CD19BiTE at the dose of 5×10^12^gc/kg. After 4 weeks, the liver, heart, spleen, lung, kidney, and brain were collected for RT-qPCR analysis to determine the contents of AAV-CD19BiTE (n=3). (B) The comparison of the ratios of activated T cells after co-culture with serum collected from AAV-GFP/CD19BiTE infused mice (n=3). (C) The changes of the contents of CD19BiTE in vivo. P value was calculated using two tailed Student’s *t*-test. |
| **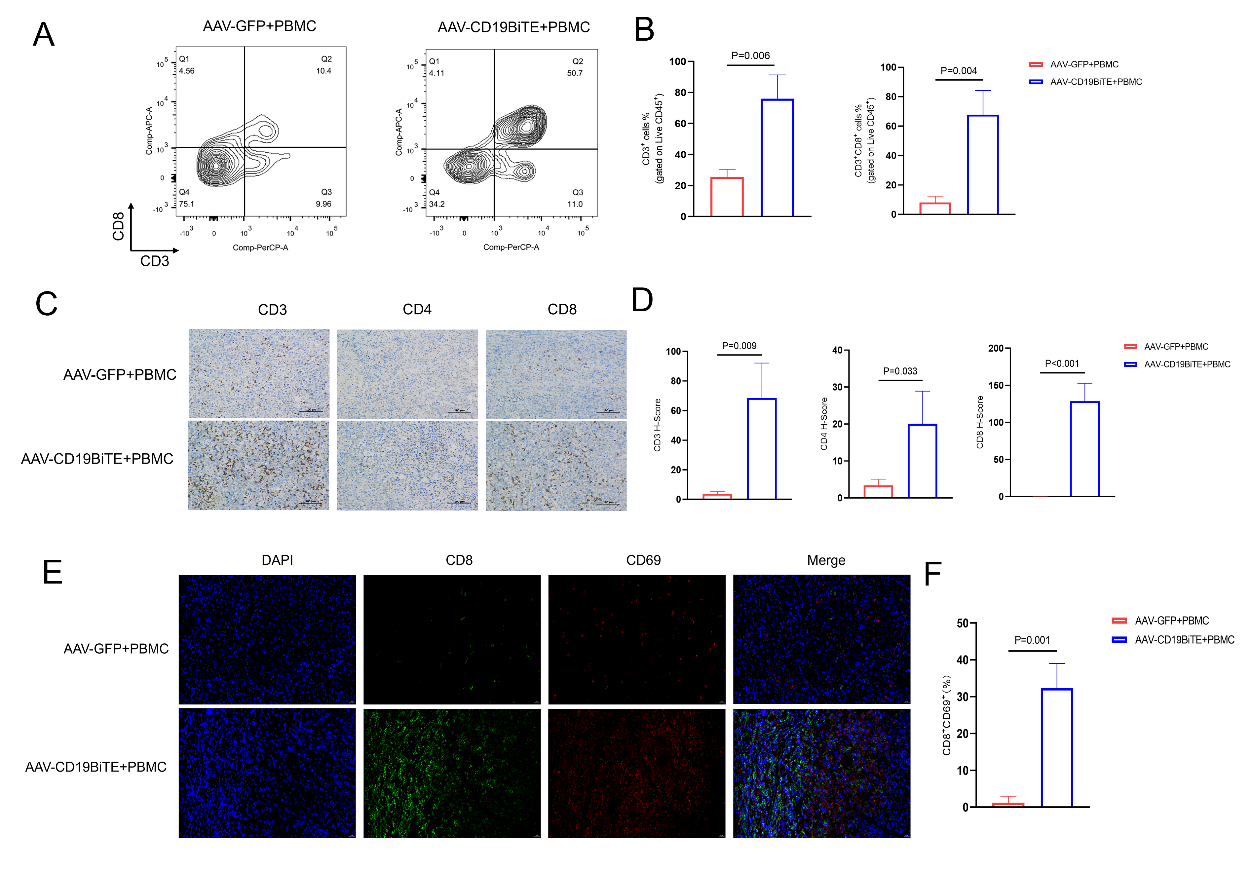** |
| **Fig. S3. Lymphoma microenvironment analyses.** (A) Flowcytometry analysis of the ratios of CD3^+^ and CD8^+^T cells in AAV-GFP and AAV-CD19BiTE groups. (B) Comparison the ratios of CD3^+^ and CD8^+^T cells in AAV-GFP and AAV-CD19BiTE groups. (C) Immunohistochemistry analysis of CD3^+^, CD4^+^, and CD8^+^T cells in AAV-GFP and AAV-CD19BiTE groups. (D) H-Scores comparison of CD3^+^, CD4^+^, and CD8^+^T cells in AAV-GFP and AAV-CD19BiTE groups. (E) Representative confocal images of immunofluorescence analysis of CD8^+^CD69^+^T cells in lymphoma treated with AAV-GFP or AAV-CD19BiTE. (F) The ratios comparison of CD8^+^CD69^+^T cells in mice treated with AAV-GFP or AAV-CD19BiTE. P values were calculated using two tailed Student’s *t*-test. |

| **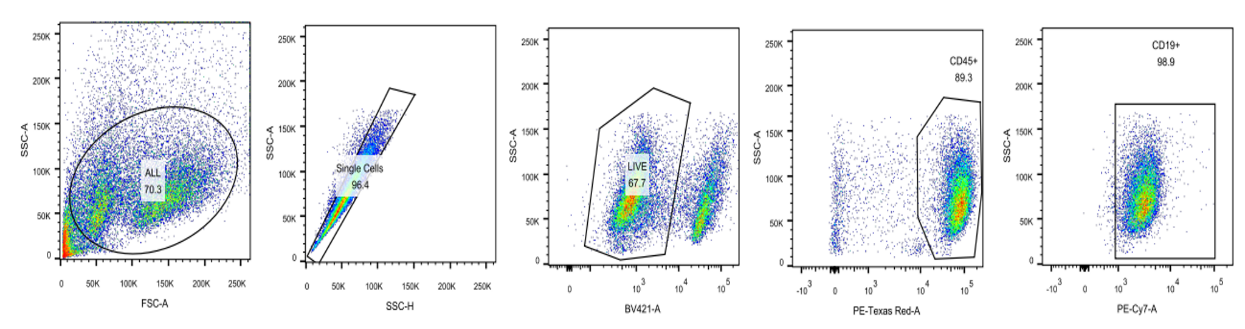** |
| --- |
| **Fig. S4. The flowcytometry analysis of tumor tissues deriving from PDX model.** The tumor tissues from constructed PDX model were collected for flowcytometry analysis, and the results proved that we established the PDX model successfully.   \| 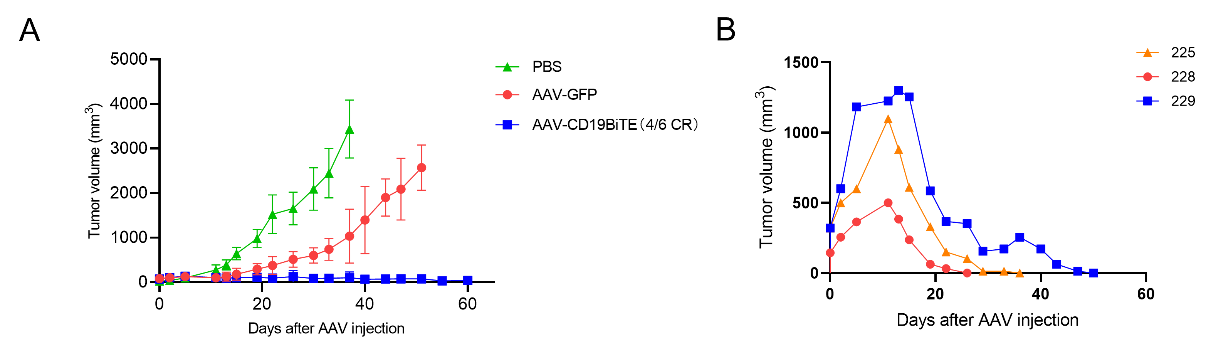  **Fig. S5. The long-term observation of the changes of tumor volumes in PDX model.** (A) Although the growth of tumor volumes was slightly slower in mice of AAV-GFP group compared to PBS group in a short time, the tumor volumes still continued to increase until similar to PBS group in a long-term observation. Conversely, the mice’s tumor volumes of AAV-CD19BiTE group began to decline after a slight increasing following AAV-CD19BiTE injection (n=6). (B) The changes of tumor volumes of mice with high tumor burden at the beginning of AAV-CD19BiTE injection. \| \| --- \| |
| **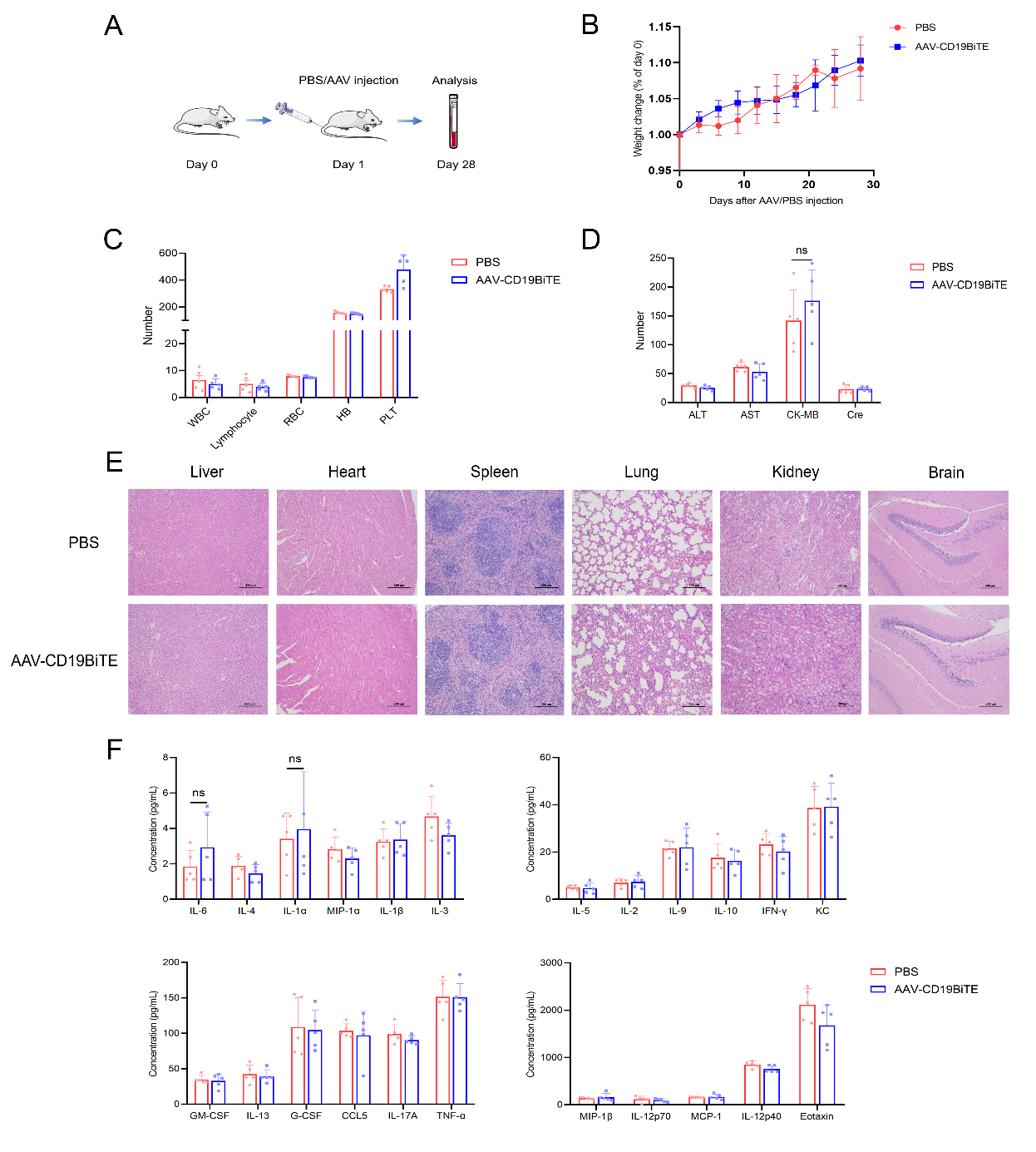** |
| **Fig. S6. Safety analysis of AAV-CD19BiTE.** (A) Balb/c mice were injected with PBS/AAV-CD19BiTE and blood were collected for safety analysis after 4 weeks. (B) The weight changes of differently treated mice. (C) The blood cell counts after PBS/AAV-CD19BiTE injection. WBC, white blood cells; RBC, red blood cells; HB, hemoglobin; PLT, platelet. (D) The biochemical indicators following PBS/AAV-CD19BiTE infusion. ALT, alanine transaminase; AST, alanine transaminase; CK-MB, creatine kinase-MB; Cre, creatinine. (E) Hematoxylin-eosin staining analysis of liver, heart, spleen, lung, kidney, and brain of differently treated mice. (F) The contents of 23 cytokines following PBS/AAV-CD19BiTE infusion. MIP-1α, macrophage inflammatory protein-1α; KC, keratinocyte-derived chemokine; GM-CSF, granulocyte-macrophage colony stimulating factor; G-CSF, granulocyte colony stimulating factor; CCL5, C-C chemokine ligand 5; MIP-1β, macrophage inflammatory protein-1β; MCP-1, monocyte chemotactic protein-1. P values were calculated using two tailed Student’s *t*-test. |

**References**

[1] Liu N, Song J, Xie Y, Wang X-L, Rong B, Man N, et al. Different roles of E proteins in t(8;21) leukemia: E2-2 compromises the function of AETFC and negatively regulates leukemogenesis. Proc Natl Acad Sci U S A. 2019;116:890-9.

[2] Marikar FMMT, Ma D, Ye J, Tang B, Zheng W, Zhang J, et al. Expression of recombinant Anti-Human FADD, preparation of its polyclonal antiserum and the application in immunoassays. Cell Mol Immunol. 2008;5:471-4.

[3] Lorenzo-Herrero S, Sordo-Bahamonde C, Gonzalez S, López-Soto A. CD107a Degranulation Assay to Evaluate Immune Cell Antitumor Activity. Methods Mol Biol. 2019;1884:119-30.

[4] Quah BJC, Warren HS, Parish CR. Monitoring lymphocyte proliferation in vitro and in vivo with the intracellular fluorescent dye carboxyfluorescein diacetate succinimidyl ester. Nat Protoc. 2007;2:2049-56.

[5] Paschalis A, Sheehan B, Riisnaes R, Rodrigues DN, Gurel B, Bertan C, et al. Prostate-specific Membrane Antigen Heterogeneity and DNA Repair Defects in Prostate Cancer. Eur Urol. 2019;76:469-78.

[6] Xu-Monette ZY, Xiao M, Au Q, Padmanabhan R, Xu B, Hoe N, et al. Immune Profiling and Quantitative Analysis Decipher the Clinical Role of Immune-Checkpoint Expression in the Tumor Immune Microenvironment of DLBCL. Cancer Immunol Res. 2019;7:644-57.

[7] Li X-Y, Wu J-C, Liu P, Li Z-J, Wang Y, Chen B-Y, et al. Inhibition of USP1 reverses the chemotherapy resistance through destabilization of MAX in the relapsed/refractory B-cell lymphoma. Leukemia. 2023;37:164-77.

[8] Lei W, Ye Q, Hao Y, Chen J, Huang Y, Yang L, et al. CD19-targeted BiTE expression by an oncolytic vaccinia virus significantly augments therapeutic efficacy against B-cell lymphoma. Blood Cancer J. 2022;12:35.

[9] Vaisitti T, Arruga F, Vitale N, Lee T-T, Ko M, Chadburn A, et al. ROR1 targeting with the antibody-drug conjugate VLS-101 is effective in Richter syndrome patient-derived xenograft mouse models. Blood. 2021;137:3365-77.
